# Supplementary material for: Causal Relationship between Aging and Anorexia Nervosa: A White-Matter-Microstructure-Mediated Mendelian Randomization Analysis
Source: Biomedicines. 2024 Aug 16;12(8):1874. doi: 10.3390/biomedicines12081874 (PMC11351342; doi:10.3390/biomedicines12081874)
Supplement: Supplementary file 1 [file biomedicines-12-01874-s001.zip › Supplementary Figure/Figure S8/Leaveoneout plot of MR analysis for TL to ubm-b-1992.pdf]

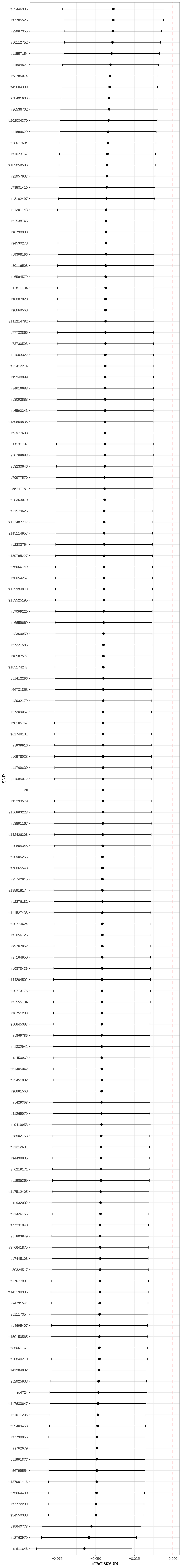

rs35446936
rs7705526
rs2967355
rs10112752
rs11557154
rs11584821
rs3785074
rs45604339
rs78491606
rs6536702
rs202034370
rs11699829
rs28577594
rs1023767
rs182059588
rs1957937
rs73581418
rs8102497
rs1291143
rs2538745
rs6790988
rs4530278
rs9398196
rs80116508
rs6584579
rs871134
rs6007020
rs6669563
rs141214782
rs77732866
rs73730598
rs1003322
rs12412214
rs9940099
rs4616688
rs3093888
rs6590343
rs139669835
rs2977608
rs131797
rs10768683
rs13230646
rs79977579
rs55747751
rs28363070
rs11579626
rs117407747
rs145114957
rs2282764
rs139795227
rs76666449
rs6054257
rs112394943
rs113525195
rs7099229
rs6659669
rs12369950
rs7221585
rs6587577
rs185174247
rs11412296
rs66731853
rs12932179
rs7209057
rs8105767
rs61748181
rs939916
rs16978028
rs11769630
rs11085072
All
rs2293579
rs116863223
rs3891167
rs142426306
rs10805346
rs10905255
rs76065543
rs5742915
rs188918174
rs2276182
rs111527438
rs10774624
rs2056726
rs3767952
rs7164950
rs9878436
rs144204502
rs10773176
rs2555104
rs6751209
rs10845387
rs869785
rs1332941
rs450962
rs61405042
rs12451892
rs6881568
rs429358
rs41269079
rs9419958
rs28502153
rs11212631
rs4498805
rs76219171
rs10885369
rs117512405
rs932002
rs11426156
rs77231040
rs17803849
rs376641875
rs17445108
rs80324517
rs17677991
rs143190905
rs4731541
rs11117354
rs4695407
rs150150565
rs56061761
rs10840270
rs41304832
rs12925933
rs4724
rs117630647
rs1611236
rs59409453
rs7790856
rs762679
rs11991877
rs56799554
rs137901416
rs75664430
rs7772289
rs34550383
rs35640778
rs2763979
rs611646
SNP
-0.075
-0.050
-0.025
0.000
Effect size (b)
